# Less Is More: Evaluating the Benefits of Minimally Invasive Spinal Surgery

**DOI:** 10.3390/life15010008

**Published:** 2024-12-25

**Authors:** Ali A. Mohamed, Rakan Alshaibi, Steven Faragalla, Garrett Flynn, Asad Khan, Emma Sargent, Youssef Mohamed, Camberly Moriconi, Cooper Williams, Zev Karve, Daniel Colome, Phillip Mitchell Johansen, Brandon Lucke-Wold

**Affiliations:** 1 Charles E. Schmidt College of Medicine, Florida Atlantic University, Boca Raton, FL 33431, USA; 2 College of Engineering and Computer Science, Florida Atlantic University, Boca Raton, FL 33431, USA; 3 Herbert Wertheim College of Medicine, Florida International University, Miami, FL 33199, USA; 4 College of Osteopathic Medicine, Kansas City University, Joplin, MO 64106, USA; 5 Department of Neurosurgery and Brain Repair, University of South Florida, Tampa, FL 33620, USA; 6 Lillian S. Wells Department of Neurosurgery, University of Florida, Gainesville, FL 32611, USA

**Keywords:** endonasal cervical, transoral cervical, transcervical, percutaneous, mini-open, tubular

## Abstract

This review aims to explore the evolution, techniques, and outcomes of minimally invasive spine surgery (MISS) within the field of neurosurgery. We sought to address the increasing burden of spine degeneration in a rapidly aging population and the need for optimizing surgical management. This review explores various techniques in MISS, drawing upon evidence from retrospective studies, case series, systematic reviews, and technological advancements in neurosurgical spine treatment. Various approaches, including endonasal cervical, transoral cervical, transcervical, mini-open/percutaneous, tubular, and endoscopic techniques, provide alternatives for current approaches to a range of spinal pathologies. The main findings of this review highlight potential advantages of MISS over traditional open surgery, including reduced complications, shorter hospital stays, and improved patient outcomes. Our research underscores the importance of adopting MISS techniques to optimize patient care in neurosurgical spine treatment.

## 1. Introduction

Spine surgery, situated at the intersection of neurosurgery and orthopedics, has evolved significantly over the years, becoming a critical and dynamic field. The prevalence of spine degeneration has seen a substantial rise in recent years, with an increasing global burden attributed to factors such as a rapidly aging population and rising obesity rates [1,2,3]. Projections of further increases in obesity rates and an aging population make it imperative to discuss advancements in spine surgery treatment for this growing patient population. With over 900,000 spinal surgeries in the US each year, constant refinement is needed to optimize this field [4,5].

The most common indications for spinal surgery include lumbar disc herniation, spinal stenosis, and degenerative spondylolisthesis [6]. Both the number of spinal segments afflicted by degeneration and the degree of degeneration per segment increase with age, with over 90% of discs being affected in patients aged over the age of 51 [7]. As is the case throughout the history of medicine, the treatment of disease states requires constant evidence-based modification and innovative problem-solving to optimize patient care. The field of spine surgery is no different, demanding an even higher threshold for change to offer a more curative measure towards pain alleviation and quality of life. These are the goals emphasized in minimally invasive spine surgery (MISS). MISS aims to successfully treat pathology while maximally preserving tissue, blood, and functionality. MISS accomplishes this through small incisions, sized slightly greater than the diameter of tools used for surgical site access. Compared with the larger incisions utilized in open surgery, this approach provides the benefit of reducing major complications from unintentional damage while decreasing reoperation rates, shortening hospital stays, and reducing rehabilitation costs [8]. Building a profound, contemporary understanding of the available approaches advances the treatment algorithms available for use and paves the way for a more robust field of neurosurgical spine treatment.

The purpose of this paper is to provide a comprehensive review of MISS, with a particular focus on its techniques, advancements, and clinical outcomes. The discussion is organized into key sections that explore various approaches and their evolving role in treating spine conditions. These include approaches such as endonasal cervical, transoral cervical, transcervical, mini-open and percutaneous, tubular, and endoscopic. By covering these distinct techniques, this review will provide a well-rounded understanding of the current state of minimally invasive spine surgery. This paper aims to provide a clear description of complex concepts involving anatomy, tools, and procedures and presents the utility of the presented techniques, which have been validated in clinical settings. By drawing upon a body of literature examining both historical developments and contemporary innovations, this paper also seeks to highlight the evolution of MISS and its role in the treatment of spine pathology. A deep understanding of MISS techniques and their indications will not only inform current treatment strategies but also help shape the future of spine surgery, ensuring that it remains adaptive to the needs of an aging and increasingly diverse patient population.

## 2. Endonasal Cervical Approaches for Pathologies of the Upper Cervical Spine

Historically, anterior approaches to the ventral spine have involved open surgical procedures. Access to this area in a minimally invasive procedure is particularly difficult due to the surrounding anatomy. Prior to the endoscopic endonasal approach (EEA), the transoral approach was widely used to access the craniovertebral junction (CVJ) [9]. The transoral-transpharyngeal approach to the CVJ risks tongue retraction damage, palate splitting, upper airway swelling, postoperative oral flora contamination, prolonged intubation, and delayed refeeding [10,11]. The EEA has now largely replaced the transoral approach for the resection of extradural lesions and odontoidectomies for its reduced risk to the patient.

The EEA first gained popularity in the 1970s for pituitary tumor resection, and its use has since been expanded to treat CVJ pathologies as well as sellar and parasellar masses, olfactory groove meningiomas, tuberculum meningiomas, craniopharyngiomas, petrous apex tumors, and clival tumors [12]. Studies have described the use of the EEA to reach the ventral cranio-cervical junction for odontoidectomy, and, in 2005, the first case report of a completely endonasal approach for the resection of the odontoid process was achieved with no significant complications [13].

In terms of minimally invasive spine surgery, the EEA is now the standard to perform odontoidectomies to treat basilar invagination due to C1–C2 facet instability, Chiari malformation, congenital defect, or degenerative processes causing irreducible ventral cervical-medullary compression as seen with rheumatoid arthritis [14,15,16]. There is also evidence to suggest the EEA is suitable for the treatment of CVJ malformation with platybasia [16,17]. The procedure feasibility is first determined by assessing the patient’s anatomical lines [18]. The EEA is preferred if the lesion is located above the palatine line, which is measured using the plane of the hard palate in the direction of the CVJ in a sagittal view [19,20]. The inferior boarder of the technique can be determined using the nasoaxial line, which is drawn from the midpoint, between the rhinion and the anterior nasal spine, towards the posterior edge of the hard palate and CVJ [21,22]. The nasoaxial and rhinopalatine line have also been documented as alternatives to the nasopalatine line to determine the inferior limit of the surgical corridor with increased accuracy [16,23,24].

Generally, the patient is positioned supine in a Mayfield retractor for stabilization, and a binostril approach with the endoscope in the right nostril and the working instrument on the left is used [22]. Next, nasopharyngeal fascia is exposed, and septoplasty and low posterior septectomy are performed. Turbinates can be resected to expand access [16]. An inverted U-shaped or linear midline incision is made in the posterior pharyngeal wall. The pharyngeal wall flap is elevated to the soft palate so that the retraction of the soft palate and subsequent expanded inferior access below the hard palate is achieved. A final dissection to identify the inferior clivus and the anterior arch is completed to reveal the finalized surgical corridor [16,22,25].

Overall, the EEA decreases hospital stays compared to the transoral approach [11,16,26,27]. It also provides minimal ventilation time and allows for earlier postoperative oral feeds compared to the transoral approach [11,25,28]. It prevents prolonged tongue retraction, the splitting of the soft or hard palates, and reduces the postoperative risks of upper airway edema, velopalatal insufficiency, and dysphagia [11,25]. Nearly 90% of patients undergoing the EEA for basal invagination experienced neurologic improvement [27,29].

Limitations of the procedure include a narrow surgical corridor and limited surgical window [25]. Suturing can also be a challenge, especially following a cerebrospinal fluid leak [19]. Risk factors of the EEA include infection, tears in the dura, vertebral artery injury, intraoperative and postoperative cerebrospinal fluid leak, and tracheotomy [11,25,27]. In conclusion, although the EEA involves a learning curve and surgical complexity, it remains a minimally invasive and effective treatment for basilar invagination, offering benefits such as reduced hospitalization and lower complication risks compared to more invasive approaches [11,17,27].

## 3. Transoral Cervical Approaches

**Figure 1:**
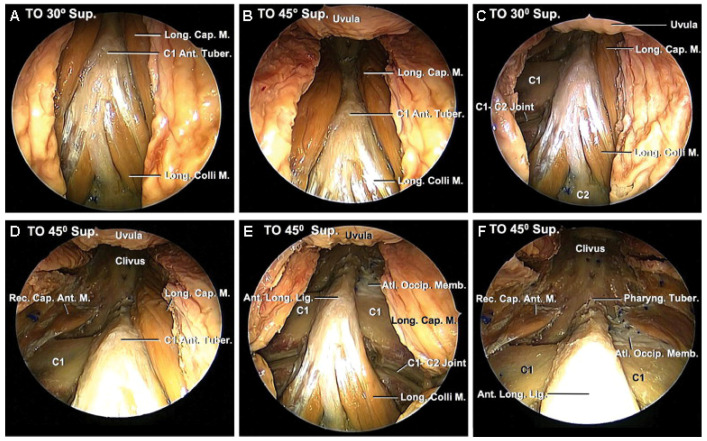
Transoral route. (**A**) 30° superior. The view extends upward behind the uvula to the upper part of the longus capitis muscles. (**B**) Closer view, 45° superior to view higher along the attachment of the longus capitis muscles to the clivus. (**C**) 30° superior. The right longus capitis muscle has been reflected laterally to expose the right half of the anterior arch of C1 and the atlantoaxial joint. (**D**) 45° upward. The attachments of the longus capitis muscles to the clivus and the rectus capitis anterior to the anterosuperior part of the occipital condyle have been exposed. (**E**) 45° superior. The longus capitis muscles have been retracted to expose the anterior atlantooccipital membrane and the attachment of the anterior longitudinal ligament to the pharyngeal tubercle. (**F**) Closer view, 45° superior. The anterior atlantooccipital membrane attaches to the anterior longitudinal ligament medially and the fibrous capsule of atlantooccipital joints laterally. The anterior longitudinal ligament ascends from the anterior surface of C1 and C2 to attach to the pharyngeal tubercle. Ant., anterior; Atl., atlantal; Cap., capitis; Lig., ligament; Long., longitudinal, longus; [42]. (© 2010. The Authors. *World Neurosurgery* published by Elsevier).

The transoral cervical approach (Figure 1) is mostly used to access lesions located behind the clivus, extending to the upper cervical spine [30,31,32,33,34,35,36]. Initially developed in the early 1900s, its popularity grew later with advancements in technology such as the operating microscope, microsurgical instruments, and retraction systems, allowing for more precise procedures [37,38,39,40]. While the transoral cervical approach remains an effective technique for accessing the retroclival region, it is now less commonly used compared to other methods like the anterolateral approach, which is favored for broader indications across the cervical spine [30]. Therefore, the transoral approach is largely reserved for specific, less common conditions in the upper cervical spine, including tumor resections, decompression surgeries, congenital abnormalities, trauma, and infection management [37,41].

The transoral cervical approach is particularly useful for accessing areas in the inferior skull and superior cervical spine that are otherwise difficult to reach. Similarly, traumatic injuries, such as odontoid fractures, often benefit from this approach due to its ability to provide better visibility and access for surgical treatment [33,34,36,37]. The transoral approach may be utilized in the resection of tumors in the craniocervical junction, upper cervical spine, and laryngopharyngeal region, where direct access is crucial for complete removal. In cases such as basilar invagination, where the cervical spine pushes into the base of the skull, the transoral approach may allow for direct visualization and access to treat compression or instability in these regions [30]. While the approach is still occasionally employed for rheumatoid arthritis-related compression or instability in the upper cervical spine, its overall use has diminished with the advent of alternative techniques [43].

The most standard and common approach to the cervical spine is using the Smith Robinson transoral cervical approach. The patient lies in the supine position with the neck slightly extended or hyper-extended. Surgeons can choose to use a retractor or fixation system to clear the surgical site of nearby structures and make an appropriate incision based on the case [33,36]. New technologies are constantly being developed and implemented in the transoral cervical approach. Technologies such as the transoral exoscope combined with the O-Arm assisted approach have been studied as a new addition to the transoral cervical approach that can allow greater visualization to surgeons in complicated cases. However, this approach is time-consuming and can be difficult to manage [44]. In addition, the endoscope-assisted transoral-transpharyngeal approach has been shown to be promising in decompression cases [45,46].

The transoral approach is not immune to complications including infection, CSF leaking, spinal instability, and damage to nearby structures [30,47,48]. With the advancement of technologies and understanding of pathophysiology, the approach risk of complications has been reduced. In a study examining the morbidity and mortality associated with the transoral cervical approach, 21.4% of patients experienced postoperative complications, and 2.4% died as a result of the surgery [49]. The study identified that the duration of the procedure was the primary contributor to postoperative complications [49]. Additionally, postoperative infections are a high concern in the transoral cervical approach, in some cases requiring debridement and a flap coverage of the pharyngeal wall [48].

## 4. Transcervical Approaches

**Figure 2:**
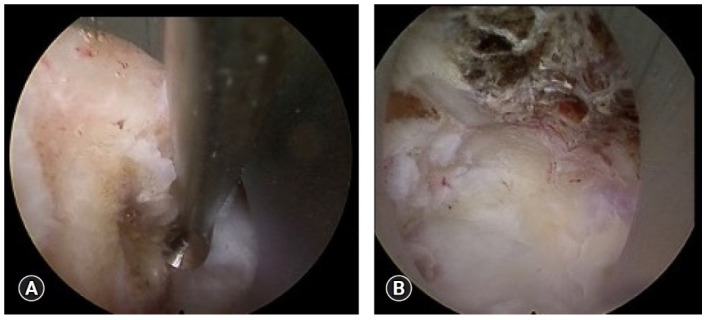
Intraoperative views step by step. (**A**) The right side of the bone edge was confirmed directly by a curved curette. (**B**) The odontoid tip and dorsal wall of the dens were resected [58]. (© 2023. The Authors. *Journal of Minimally Invasive Spine Surgery and Technique*).

**Figure 3:**
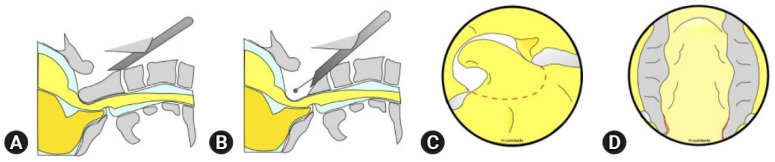
(**A**,**B**) Schema of the lateral view. (**C**) Drilling area. (**D**) After cyst resection. Pulsation of the soft tissue was confirmed [52]. (© 2023. The Authors. *Journal of Minimally Invasive Spine Surgery and Technique*).

Transcervical approaches are increasingly used to treat pathologies at the craniovertebral junction (CVJ), such as odontoid abnormalities and tumors (Figure 2 and Figure 3), with the help of intraoperative CT and 3D stereotactic navigation technology [50,51]. Though this approach was initially met with skepticism due to concerns over limited visualization and proximity to critical neurovascular structures, advancements in technology have significantly enhanced its feasibility and safety. Today, transcervical MISS is effective for pathologies such as degenerative disc disease, cervical stenosis, basilar invagination, and certain tumors, especially when traditional open procedures are challenging [51].

Transcervical MISS is primarily indicated for pathologies requiring anterior decompression at the CVJ, such as odontoid fractures, pseudotumors, and basilar impression. Traditionally, posterior decompression with occipitocervical fixation was used for these conditions, but the anterior approach allows for direct access to ventral structures without the limitations of the open transoral method, which is hindered by poor visibility and a higher rate of complications [51]. The advent of full-endoscopic uniportal transcervical odontoidectomy has revolutionized the management of ventral CVJ pathologies. This technique allows for targeted odontoidectomy with minimal disruption, using a small 4 cm incision and fluoroscopy-guided navigation systems. In a series of 12 patients, this method showed improved outcomes for conditions like basilar impression and pseudotumors, with no significant complications or blood loss [51]. Further endoscopic transcervical approaches for image-guided odontoidectomy have been described for symptomatic irreducible basal invagination resulting from the over-projection of the odontoid process [52]. Full-endoscopic uniportal decompressions can also be performed retropharyngeally with similar expected outcomes [53]. Retro-odontoid pseudotumors, such as pannus, can impinge on the spinal cord and nerves, leading to severe neck pain, cervicomedullary compression, and myelopathy. These conditions can be treated with ventral or dorsal decompression, along with posterior fixation [54]. Additionally, unilateral laminotomy for bilateral decompression (ULBD) provides a minimally invasive approach for treating cervical stenosis, offering the benefit of preserving paravertebral muscle function, which aids in postoperative recovery [55]. Conditions such as cervical spondylotic myelopathy (CSM) can also be addressed using minimally invasive techniques like anterior cervical discectomy and fusion (ACDF), performed through tubular retractors and fluoroscopic control [56,57].

In the traditional transcervical approach, the anterior cervical spine is typically accessed through a small incision below the mandible with careful displacement of soft tissues to expose the surgical field. This approach can be utilized in pathologies of multiple vertebral levels as it conveys direct access to the ventral cervical spine to facilitate decompression, instrumentation, and fusion as needed. For full-endoscopic uniportal transcervical endoscopic odontoidectomy, a transverse incision is made at the C4 level with resection of the odontoid using an endoscopic burr. Navigation-assisted techniques, such as intraoperative fluoroscopy, ensure precision during decompression and instrumentation [52]. Decompression is performed by removing the ligamentum flavum attachments, superiorly followed by partial medial facetectomy to expose the dura and nerve root [59]. For cervical endoscopic ACDF, serial dilation is employed to expose the herniated disc under endoscopic guidance, and a spinal cage is implanted using an endoscopic cage holder [56,59].

Research on minimally invasive techniques to the transcervical approach has demonstrated several advantages including minimal blood loss, reduced muscle disruption, shorter hospital stays, and quicker recovery compared to traditional open procedures [34,60,61]. Furthermore, postoperative pain is lower, leading to reduced narcotic use and improved cosmetic outcomes thanks to smaller incisions [34]. However, while transcervical MISS does not significantly outperform traditional open techniques in terms of long-term clinical outcomes, it offers clear benefits in fusion cases, with shorter hospital stays and lower revision and readmission rates [62]. Despite its advantages, transcervical MISS is not free of risks. Complications include radiculitis, incidental durotomy, and the malposition of screws [63]. Interestingly, the rate of incidental durotomy in cervical surgeries is lower (1.4%) compared to thoracic (3.8%) and lumbar (7.8%) surgeries [64].

## 5. Mini-Open/Percutaneous

**Figure 4:**
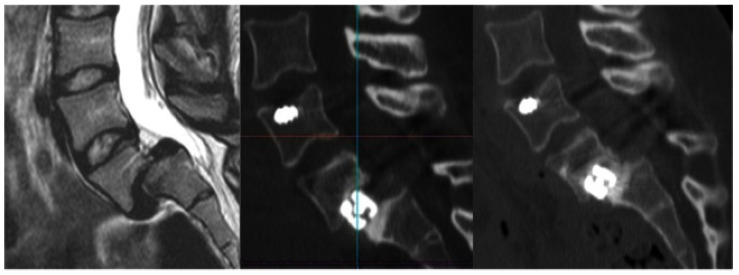
Preoperative spondylolisthesis L5–S1 grade II–III (**Left**). Postoperative reduction after Opticage™ expansion and posterior screw fixation (**Middle**). Evidence of bony fusion 24 months after surgery (**Right**) [65]. (© 2015. The Authors. *International Journal of Spine Surgery*).

Mini-open and percutaneous spine surgery are separate but similar minimally invasive surgical techniques, and the terms are often used interchangeably (Figure 4). Mini-open spine surgery involves smaller incisions and a reduced disruption of the musculature in comparison to traditional open surgery. Percutaneous spine surgery is characterized by the use of needle puncture and small skin incisions, epitomizing the minimization of surgical invasiveness. This approach heavily relies on imaging techniques for guidance.

Both mini-open and percutaneous procedures have gained popularity as spine surgery has evolved towards minimally invasive techniques [66]. These methods are particularly effective for treating lumbar disorders such as disc herniation, lumbar spinal stenosis, or spondylolisthesis [33,67]. Their minimal invasiveness offers significant advantages over traditional surgical approaches, including decreased blood loss and shorter hospitalizations [68,69].

A notable advancement in this field is the Minimally Invasive Transforaminal Lumbar Interbody Fusion (MIS-TLIF), which has become increasingly preferred for managing conditions like degenerative disc disease and spinal instability. MIS-TLIF provides a less invasive alternative to open-TLIF, aligning with the current trend towards reducing surgical trauma while maintaining efficacy [70]. Percutaneous osteosynthesis is also being used in the treatment of degenerative spinal diseases and has gained prevalence in trauma care, often as the primary treatment for non-neurological spinal fractures and in polytrauma cases [71,72]. Mini-open and percutaneous procedures are also being considered for metastatic spine disease, where they have been associated with shorter recovery times and reduced morbidity [73]. In the cervical region, percutaneous techniques show promise, particularly in treating cervical radiculopathy caused by conditions like spondylosis and stenosis [67]. The use of percutaneous facet joint cage implants, which involve a screw and washer mechanism, has been effective for decompression and fusion in these cases [74]. However, these treatments have not yet been established as consistently effective, and further study is needed to better understand their potential benefits.

Overall, mini-open and percutaneous procedures provide many benefits. The most cited benefits include less intraoperative blood loss, less paravertebral muscle damage, and shorter recovery times [75,76,77]. There is also some evidence supporting a lower rate of infections when comparing posterior percutaneous pedicle screw fixation vs. open surgical instrumented fusion for thoraco-lumbar palliative management [77]. It is important to note the trade-offs, which include higher radiation exposure and increased surgery costs [75,76]. A meta-analysis comparing posterior minimally invasive surgery with open TLIF/PLIF found no significant difference in operative time or adverse surgical events between the two techniques. The study highlighted that while surgical complications were similar, medical complications were lower in the minimally invasive group. Specifically, operative time was not significantly decreased, and there was no difference in nonunion or reoperation rates. However, the meta-analysis indicated decreases in estimated blood loss, time to ambulation, and the length of stay in the MISS group. Furthermore, medical adverse events were less likely [78]. Comparative studies in traumatic spinal fractures of the thoracic and lumbar spine have shown a reduction in complication rates from 14.8% in open surgical approaches to 5.3% in percutaneous approaches [79]. Additionally, for traumatic spine injuries requiring dorsal stabilization, percutaneous and open methods showed similar effectiveness in fracture reduction but with fewer severe complications and no prolonged operation time in the percutaneous approach [80]. In summary, mini-open and percutaneous spine surgeries present a favorable balance of reduced invasiveness, quicker recovery, and lower complication rates despite certain challenges such as increased radiation exposure and specific technical concerns. These findings underscore the nuanced advantages of MISS in modern spinal treatment.

## 6. Tubular

**Figure 5:**
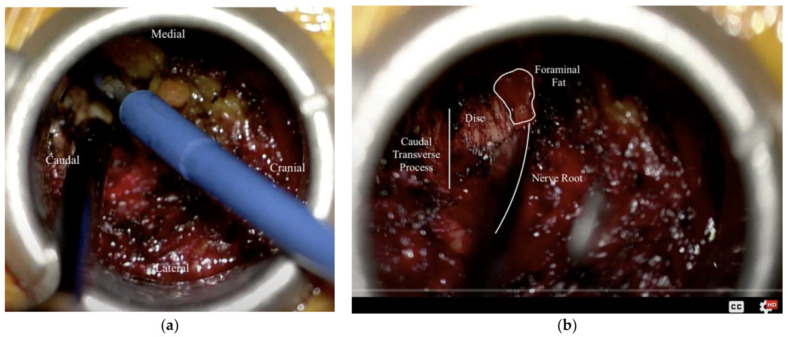
Intra-operative photo from a right-sided (ipsilateral) extra-foraminal approach to the L4–5 FLDH. (**a**) Exposure of the caudal transverse process and facet junction using the Bovie electrocautery; (**b**) following the partial removal of the overlying intertransverse membrane attached to the caudal transverse process, immediately exposing the disc space, allowing for further exploration of the foramen (fat contained within the foramen is highlighted) as well as the exiting nerve root without need for complete stripping of overlying soft tissue [91]. (© 2022. The Authors. *Medicina* published by MDPI).

Tubular retraction systems were first implemented in 1977 for lumbar discectomies, marking an important advancement in minimally invasive spinal surgeries [81]. Prior to the implementation of the Minimal Exposure Tubular Retractor (METRx) system, speculum or polyethylene tubes were used as makeshift dilator systems and have since been adapted to cover the entire neuroaxis with comparable outcomes to more invasive techniques [82,83,84]. These systems have also been adapted for use in brain tumors, highlighting their specificity and precise nature [85]. In 2003, a further advancement of this technique was made when a microscope was integrated, coining the term “microtubular discectomy” [86,87]. From this advancement, it has become the gold standard for decompressing lumbar spinal stenosis while effectively minimizing paraspinal muscular and ligament damage (Figure 5) [88,89]. One of the main benefits of reducing surrounding tissue damage is the reduction of scar tissue formation, allowing for reentry at the site of the initial operation for potential revisions [90].

Specific indications for use of tubular retractor systems include discectomies, spinal fusions, and even the removal of pituitary adenomas [92,93,94]. Paramedian incisions are made, and two dilator tubes are inserted until adequate space is present for the tubular retractor system to be inserted [95,96]. The first dilator tube is used to maintain the incision space, while the second dilator tube is used to widen the view of the targeted area of the spinal column on a subcutaneous level [97]. This double tubular system greatly increases the viewing window for operative ease [97]. The dura is tacked bilaterally to maintain this window, and, in the case of the resections of tumor removals, bayonetted instruments are able to be inserted through and retract the tissue until an appropriate window is formed for dissection [98,99].

As far as outcomes are concerned, multiple studies have been conducted to determine the safety and efficacy of tubular versus open surgical techniques. It has been found that decreased post-operative drainage is required with tubular techniques, and the time of drainage is also significantly decreased [100]. For discectomies, equivalent operating times with lower blood loss and shorter hospital stays were identified when compared to standard discectomies [81]. A separate study carried out focusing on posterior cervical laminoforaminotomy operations showed tubular retractor systems having excellent or good results in 97% of patients [101]. When compared to open operations, the tubular technique has demonstrated advantages including reduced blood loss and shorter hospital stays, which has contributed to overall lower healthcare costs [102,103].

## 7. Endoscopic

Endoscopic spine surgery (ESS) is a minimally invasive surgery technique that utilizes an endoscope visualization of the surgical site [104]. Technological advancements, including high-resolution cameras, improved light sources, and high-speed burrs, have enabled more complex spinal surgeries to be performed endoscopically [105,106,107]. Fully endoscopic spinal surgery employs either a single working channel endoscope or two ports, one for surgical instruments and the other for the camera [108].

Originally, this approach was primarily used for lumbar disc herniations; however, due to continued advancements, it is now also employed for the thoracic and cervical spine [109,110]. Lumbar ESS is commonly used for paracentral disc prolapse but can also address various disc herniation types, such as central, subarticular, and far-lateral herniations [111]. Early ESS required fluoroscopy, but recent developments have integrated navigation, robotics, and augmented reality, expanding ESS indications to include endoscopic fusion and stenosis treatment [112,113,114]. Initially, one of the primary disadvantages of endoscopic spine surgery was the lack of stereognosis and depth sensation; however, each of these issues is being addressed with navigation and augmented reality [115].

ESS can be approached via the interlaminar or extraforaminal route, and the choice of approach is often guided by the location and nature of the pathology [116]. ESS systems include percutaneous, microendoscopic, and biportal endoscopic systems [117]. Percutaneous endoscopic systems involve a stab incision and the use of a guide wire to insert the endoscope, with constant saline irrigation to minimize tissue damage [118]. Microendoscopic systems, on the other hand, use a rigid endoscope with a larger tubular retractor, allowing for the simultaneous use of multiple instruments independently of the camera [119]. Biportal endoscopy uses two ports, offering greater flexibility and enabling conventional instruments like nerve root retractors to protect surrounding tissue [120,121]. Biportal systems have gained popularity due to a shorter learning curve and lower costs, although the monoportal approach remains favored for minimizing soft tissue disruption [113,115].

The outcomes of ESS have been promising. Studies consistently report reduced postoperative pain, shorter hospital stays, less blood loss, and lower complication rates compared to traditional open surgeries [122,123,124]. The technique also minimizes soft tissue injury and allows access to difficult-to-reach anatomic areas [125]. Some studies suggest long-term motion preservation with cervical decompression and fusion using fully endoscopic approaches [126]. However, ESS can cause unique complications, which vary depending on the approach. For the anterior cervical approach, complications include difficulty swallowing, hematoma, and hoarseness, with complication rates ranging from 3.75% to 18.5% [127]. The posterior cervical approach shows a range of complication rates, from 1.15% to 33.3%, including nerve root injury and dysesthesia. Complications at the thoracic spine level include dural tears, hematoma, and incomplete decompression [128]. Reported complications in lumbar spine surgery include poor outcomes in 6% of patients undergoing lumbar ESS for herniated discs, with issues such as dural tears, hematoma, pain, and infection [129,130,131]. Additionally, anterior approaches to lumbar spine surgery are associated with higher postoperative morbidity and reoperation rates compared to posterior approaches [132,133].

## 8. Conclusions

This review provides a comprehensive examination of MISS within the context of neurosurgical practice. By reviewing the existing literature and exploring technological advancements, we have identified various MISS approaches and provided comparisons to traditional open surgery, examining factors such as surgical outcomes, complication rates, and patient satisfaction. The adoption of MISS techniques represents a key advancement in neurosurgical spine treatment, aiding current treatment modalities and providing alternative solutions for diverse spinal pathologies. Despite challenges in adoption, the growing body of evidence supports the efficacy of MISS and presents many opportunities for continued investigation and innovation. As the field continues to evolve, a comprehensive understanding of MISS techniques and outcomes will drive further advancements, ultimately improving patient and physician satisfaction while developing novel intervention strategies for neurosurgical spine surgery.

## Data Availability

All data are available within the article.
